# Berberine Inhibits Doxorubicin-Triggered Cardiomyocyte Apoptosis via Attenuating Mitochondrial Dysfunction and Increasing Bcl-2 Expression

**DOI:** 10.1371/journal.pone.0047351

**Published:** 2012-10-15

**Authors:** Xiuxiu Lv, Xiaohui Yu, Yiyang Wang, Faqiang Wang, Hongmei Li, Yanping Wang, Daxiang Lu, Renbin Qi, Huadong Wang

**Affiliations:** 1 Department of Pathophysiology, School of Medicine, Jinan University, Guangzhou, Guangdong, China; 2 Key Laboratory of State Administration of Traditional Chinese Medicine of the People’s Republic of China, School of Medicine, Jinan University, Guangzhou, Guangdong, China; Massachusetts Eye & Ear Infirmary, Harvard Medical School, United States of America

## Abstract

Cardiomyocyte apoptosis is an important event in doxorubicin (DOX)-induced cardiac injury. The aim of the present study was to investigate the protection of berberine (Ber) against DOX- triggered cardiomyocyte apoptosis in neonatal rat cardiomyocytes and rats. In neonatal rat cardiomyocytes, Ber attenuated DOX-induced cellular injury and apoptosis in a dose-dependent manner. However, Ber has no significant effect on viability of MCF-7 breast cancer cells treated with DOX. Ber reduced caspase-3 and caspase-9, but not caspase-8 activity in DOX-treated cardiomyocytes. Furthermore, Ber decreased adenosine monophosphate-activated protein kinase α (AMPKα) and p53 phosphorylation at 2 h, cytosolic cytochrome c and mitochondrial Bax levels and increased Bcl-2 level at 6 h in DOX-stimulated cardiomyocytes. Pretreatment with compound C, an AMPK inhibitor, also suppressed p53 phosphorylation and apoptosis in DOX-treated cardiomyocytes. DOX stimulation for 30 min led to a loss of mitochondrial membrane potential and a rise in the AMP/ATP ratio. Ber markedly reduced DOX-induced mitochondrial membrane potential loss and an increase in the AMP/ATP ratio at 1 h and 2 h post DOX exposure. In *in vivo* experiments, Ber significantly improved survival, increased stroke volume and attenuated myocardial injury in DOX-challenged rats. TUNEL and Western blot assays showed that Ber not only decreased myocardial apoptosis, caspase-3 activation, AMPKα and p53 phosphorylation, but also increased Bcl-2 expression in myocardium of rats exposed to DOX for 84 h. These findings indicate that Ber attenuates DOX-induced cardiomyocyte apoptosis via protecting mitochondria, inhibiting an increase in the AMP/ATP ratio and AMPKα phosphorylation as well as elevating Bcl-2 expression, which offer a novel mechanism responsible for protection of Ber against DOX-induced cardiomyopathy.

## Introduction

Doxorubicin (DOX), as a broad-spectrum antitumor antibiotic, is frequently used in chemotherapy due to its excellent anticancer efficacy [1]. Although rapid progress has been made on the optimal usage of DOX for decades, its dose-dependent and cumulative cardiotoxicity still remains a major concern [2]. DOX may induce acute and chronic cardiotoxicity leading to irreversible heart failure with high mortality, this specific cardiac dysfunction does not respond well to the usual therapy as other types of heart dysfunction. Despite extensive basic and clinical researches having continued for decades, the precise mechanisms of DOX-induced cardiomyopathy is not fully elucidated, and the presently available therapy for established cardiomyopathy has not demonstrated the expected success [3], [4]. Recently, increasing evidence suggests that cardiomyocyte apoptosis plays an important role in the DOX-induced cardiomyopathy [5]–[8]. Blockage of apoptotic pathways with over-expression of a modified bifunctional apoptosis regulator can significantly attenuate DOX-induced cardiomyocyte apoptosis and improve cardiac dysfunction [9]. Some investigations have further implicated p53 tumor suppressor protein and adenosine monophosphate-activated protein kinase (AMPK) signal activation in the DOX-triggered cardiomyocyte apoptosis, inhibition of AMPK and p53 phosphorylation can suppress DOX-stimulated cardiomyocyte apoptosis, and targeted disruption of p53 also attenuates DOX-induced cardiac injury [10]–[13]. Therefore, prevention of cardiomyocyte apoptosis may be considered as a therapeutic target for the treatment of DOX-induced cadiomyopathy.

**Figure 1:**
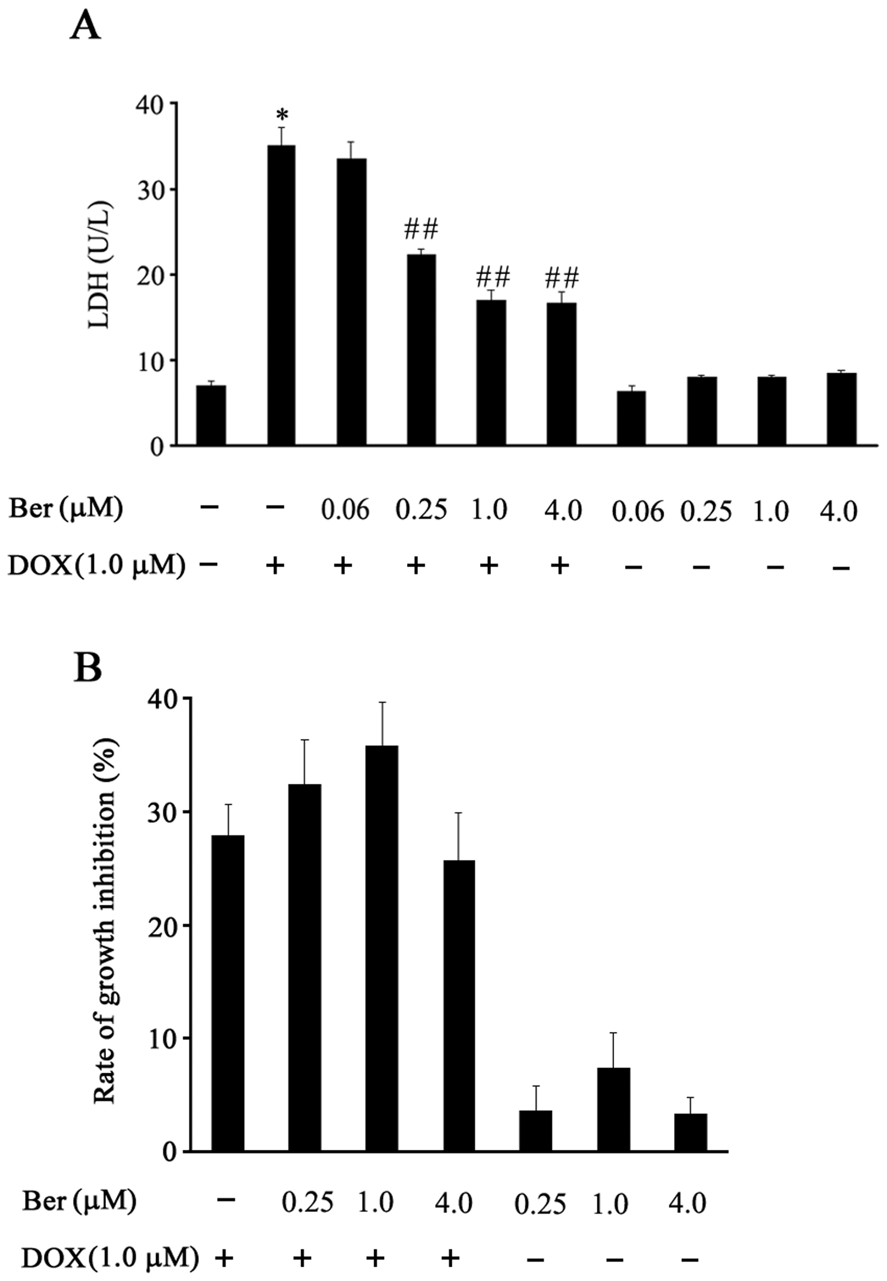
Effect of Ber on cardiomyocyte viability and MCF-7 cell growth inhibition after DOX treatment. (A) Cardiomyocytes were treated with Ber in the presence or absence of DOX for 24 h, lactate dehydrogenase (LDH) activity in the supernatants was detected (n = 4). (B)The effect of Ber on growth inhibition of MCF-7 cells treated with DOX for 24 h (n = 4). The MCF-7 cell viability was examined with the Cell Counting kit-8. *P<0.05 compared with control group. ^# #^ *P*<0.01 compared with DOX group.

Berberine (Ber) is an alkaloid from the *coptis chinensis* species. It has long history of use for treating diarrhea in traditional Chinese medicine. A growing number of studies reveal that Ber has a wide variety of biological effects, including anti-tumor and cardiovascular-protective actions [14]. Marin-Neto, et al. observed Ber improved cardiac function in patients with severe congestive heart failure [15]. Recently, Zhao, et al. reported Ber might have a potential protection against DOX-induced cardiotoxicity [16]. However, there is no investigation concerning the effect of Ber on DOX-triggered cardiomyocyte apoptosis. Our previous study has showed that Ber attenuates myocardial apoptosis and cardiac dysfunction in endotoxemic mice [17]. Thus, we hypothesized Ber could inhibit DOX-induced cardiomyocyte apoptosis. To test this possibility, we investigated the protective effect of Ber against DOX-induced cardiomyocyte apoptosis *in vitro* and *in vivo*, and further elucidated the underlying mechanism in the present study. The results showed for the first time that Ber inhibited DOX-induced cardiomyocyte apoptosis in neonatal rat cardiomyocytes. In addition, Ber also attenuated myocardial apoptosis and improved survival in rats challenged with DOX. The mechanisms responsible for this protection of Ber may be related to the protection of mitochondria, inhibition of AMPKα and p53 phosphorylation as well as increase in Bcl-2 expression.

**Figure 2:**
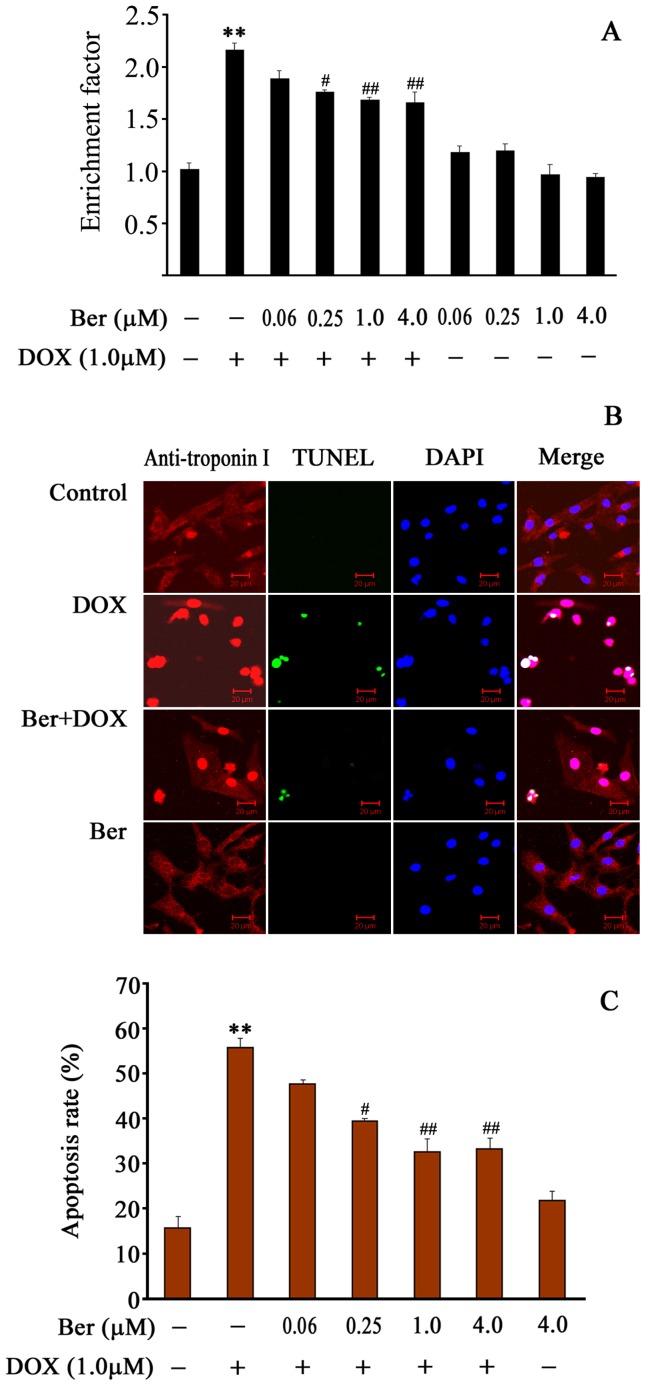
Effect of Ber on neonatal rat cardiomyocyte apoptosis induced by DOX. (A) Cardiomyocytes were treated with DOX in the absence or presence of Ber for 24 h. Apoptosis was assessed by the detection of mono- and oligonucleosomes (histone-associated DNA fragments) using a Cell Death Detection ELISA kit. The levels of mono- and oligonucleosomes in the cytoplasm of apoptotic cells were expressed as enrichment factor, which was calculated as the ratio of the absorbance of DOX or/and Ber- treated cells to absorbance of untreated control (n = 5). (B) Apoptotic nuclei were detected by TUNEL assay after Ber (4.0 µM) or/and DOX (1.0 µM) treatment for 24 h. Bars, 20 µm. (C) The apoptosis rate was expressed as the ratio of TUNEL-positive cardiomyocyte nuclei to total number of cardiomyocyte nuclei (n = 5). **P<0.01 compared with control group. ^#^ *P*<0.05, ^#^^#^ *P*<0.01 compared with DOX group.

## Methods

### Ethics Statement

All experiments in the present study were conducted in strict accordance with the recommendations in the Guide for the Care and Use of Laboratory Animals published by the US National Institutes of Health. The animal experimental protocol was previously approved by the Animal Care and Use Committee at School of Medicine, Jinan University (Permit Number: AE2011042501). All surgery was performed under sodium pentobarbital anesthesia, and every effort was made to minimize suffering.

**Figure 3:**
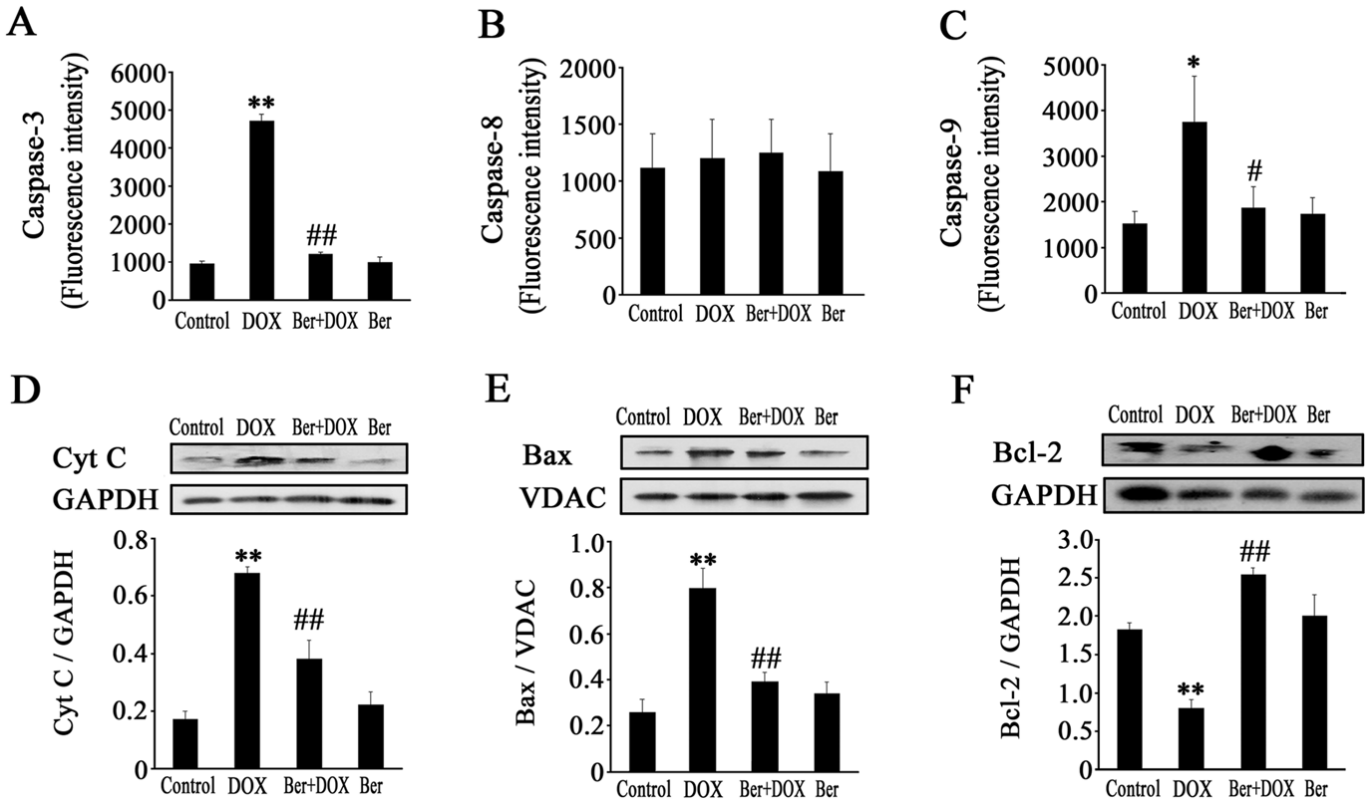
Effect of Ber on caspase activities, Bcl-2 protein level, cytoplasmic cytochrome c (Cyt c) and mitochondrial Bax contents in DOX-treated cardiomyocytes. (A, B and C) Cardiomyocytes were treated with DOX (1.0 µM) in the absence or presence of Ber (1.0 µM) for 12 h, caspase-3 (n = 4), caspase-8 (n = 4) and caspase-9 (n = 6) activity were analyzed by flow cytometry. (D, E and F) Cardiomyocytes were treated with DOX (1.0 µM) in the absence or presence of Ber (1.0 µM) for 6 h, levels of cytoplasmic Cyt c, mitochondrial Bax and Bcl-2 (whole cell homogenate) proteins were determined by Western blotting (n = 4). *P<0.05, **P<0.01 compared with control group. ^#^ *P*<0.05, ^#^^#^ *P*<0.01 compared with DOX group.

### Neonatal Rat Cardiomyocyte Culture and Treatment

The neonatal Sprague-Dawley rats (1–2 days old), obtained from the medical laboratory animal center of Guangdong province (Guangzhou, China), were deeply anesthetized with pentobarbital sodium (100 mg/kg) and decapitated for cardiac tissue harvesting, left ventricular cardiomyocytes were then enzymentically dissociated, and cultured in DMEM, supplemented with 10% fetal bovine serum, 100 U/ml penicillin and 100 µg/ml streptomycin, at 37°C in a humidified incubator with 5% CO_2_ for 48 h. Cells were treated with Ber (neutral sulfate berberine, Sigma, St. Louis, MO, USA) at concentration of 0.06, 0.25, 1.0, 4.0 µM or vehicle for 20 min, and then exposed to 1 µM DOX (Doxorubicin hydrochloride, Sigma, St. Louis, MO, USA) for indicated time. In separate experiments, cells were pre-incubated with Compound C (Cell Signaling Technology, Danvers, MA, USA), an inhibitor of AMPK phosphorylation, or 5-aminoimidazole-4-carboxyamide ribonucleoside (AICAR, Sigma, St. Louis, MO, USA), a AMPK activator, 90 min before addition of 1 µM DOX or/and 1.0 µM Ber. The same volumes of corresponding solvents were added to the controls. Additionally, In order to examine the effects of Ber and DOX at varying doses on AMPK phosphorylation in neonatal rat cardiomyocytes, cardiomyocytes were incubated with different concentrations of Ber or DOX for 2 h in independent experiments.

**Figure 4:**
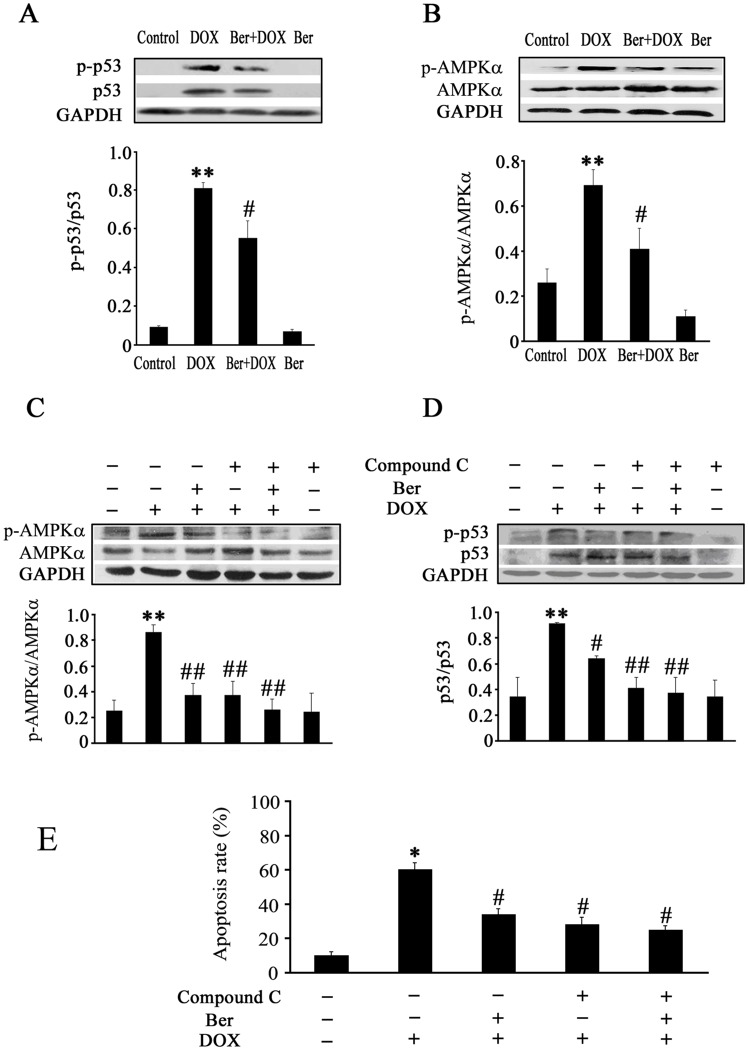
Changes in p53 and AMPKα phosphorylation as well as apoptosis rate in neonatal rat cardiomyocytes after DOX, Ber or/and compound C treatment. Cardiomyocytes were exposed to DOX (1.0 µM) or/and Ber (1.0 µM) for 2 h, p53 and p-p53 protein levels (A, n = 4) as well as AMPKα and p-AMPKα (Thr172) protein levels (B, n = 5) were detected by Western blot assay. (C and D) Compound C and Ber inhibit AMPK and p53 phosphorylation in DOX–treated cardiomyocytes (n = 5). Cardiomyocytes were pretreated with compound C (1.0 µM) or vehicle for 90 min, and then incubated with DOX (1.0 µM) or/and Ber (1.0 µM) for 2 h, AMPK and p53 phosphorylation were measured by Western blot assay. (E) Compound C and Ber decrease DOX-induced cardiomyocyte apoptosis. Cardiomyocytes were pretreated with compound C (1.0 µM) or vehicle for 90 min, and then exposed to DOX (1.0 µM) or/and Ber (1.0 µM) for 24 h, the cardiomyocyte apoptosis was measured using TUNEL assay (n = 5). *P<0.05, **P<0.01 compared with control group.^ #^ *P*<0.05, ^#^^#^ *P*<0.01 compared with DOX group.

### Cytotoxicity Assays

To measure cytotoxicity, MCF-7 breast cancer cells and neonatal rat cardiomyocytes were plated at a density of 5×10^4^ cells/well, respectively. After treatment with DOX or/and Ber for 24 h, the MCF-7 cell viability was examined with the Cell Counting kit-8 (Dojindo Molecular Technologies, Inc., Japan) as the manufacturer’s instructions. The absorbance (OD) values at 450 nm were examined, and MCF-7 cell growth inhibition rate (%) was calculated using the following equation: [(1–OD of drug-treated group/OD of control)×100%]. Cardiomyocyte cytotoxicity was assessed by measuring the release of lactate dehydrogenase (LDH) from the cells. After cardiomyocyte exposure completed, culture medium was collected and LDH activity in the supernatants was determined by biochemistry autoanalyser.

**Figure 5:**
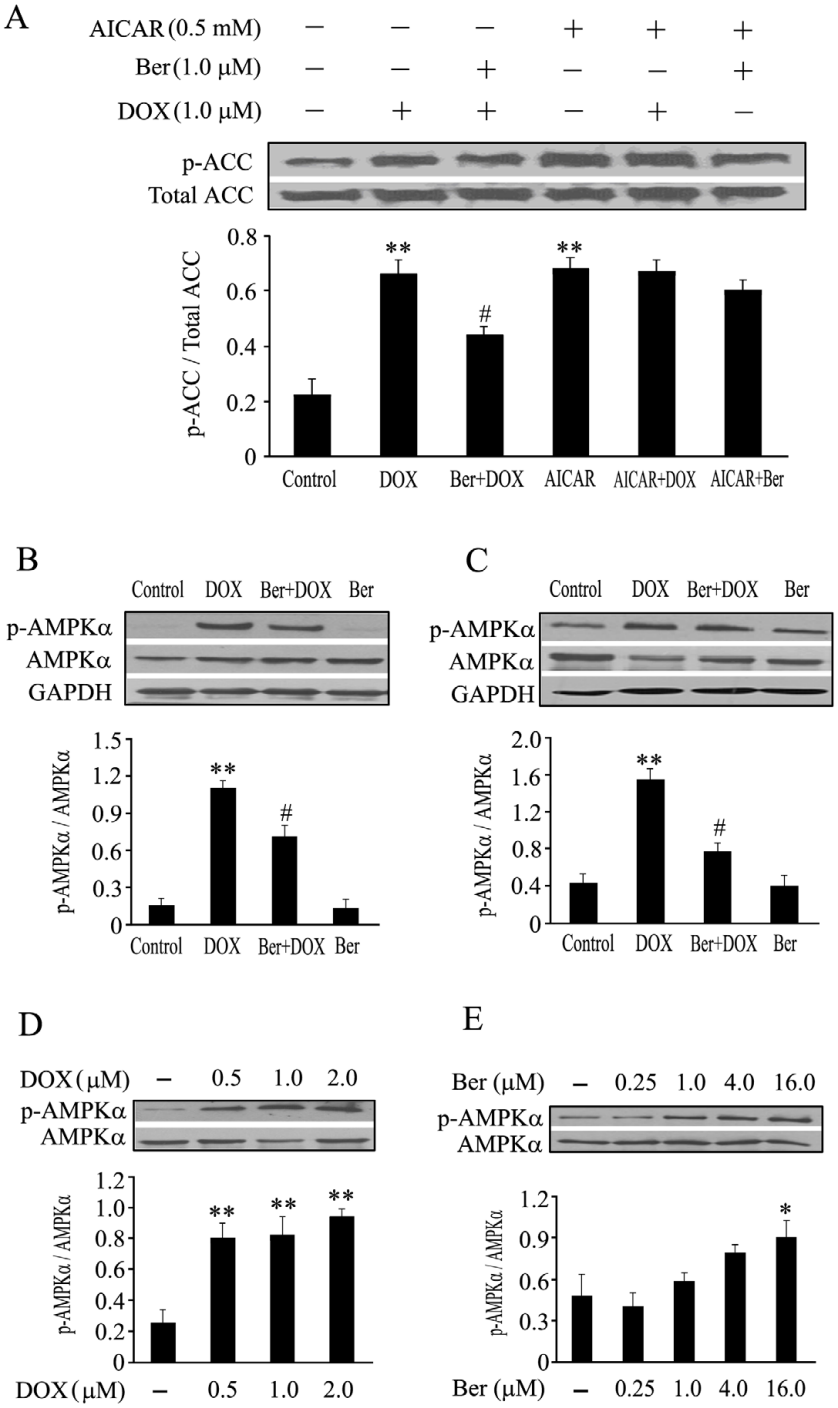
Changes in phosphorylation of acetyl-CoA carboxylase (ACC), time course and dose-dependent alterations of AMPKα phosphorylation in neonatal rat cardiomyocytes treated with DOX or/and Ber. (A) Cardiomyocytes were pretreated with AMPK activator, 5-aminoimidazole-4-carboxyamide ribonucleoside (AICAR) (0.5 mM), or vehicle for 90 min, and then incubated with DOX (1.0 µM) or/and Ber (1.0 µM) for 2 h, the Ser79 phosphorylation level of ACC, a downstream target of AMPK, was measured by Western blot assay (n = 4). (B and C) Time course of AMPKα phosphorylation. Cardiomyocytes were treated with DOX (1.0 µM) or/and Ber (1.0 µM) for 12 h (B) or 24 h (C), and AMPKα phosphorylation was examined by Western blot assay (n = 4). (D, E) Dose-dependent changes of AMPKα phosphorylation induced by DOX or Ber. Cardiomyocytes were treated with DOX (D, n = 3) or Ber (E, n = 4) at varying doses for 2 h, and Western blotting was performed to detect the AMPKα phosphorylation. *P<0.05, **P<0.01 compared with control group.^ #^ *P*<0.05 compared with DOX group.

### *In vivo* Experimental Design

The male adult (8–10 weeks old) Sprague-Dawley rats were obtained from the medical laboratory animal center of Guangdong province (Guangzhou, China) and allowed to acclimate to the new environment for 3 days prior to experiment in a standard experimental room (12 h light/dark cycle, 24°C and 50%–70% humidity) with free access to commercial standard chow and tap water. The rats were randomly assigned to the following groups: Control, DOX, DOX+Ber and Ber. These rats were intraperitoneally injected with normal saline (2 mL) or a single dose of DOX (20 mg/kg, 2 mL), followed immediately by intragastrical administration of 2 ml distill water or Ber at a dose of 30, 60 or 120 mg/kg, and then once a day for another three consecutive days. Then, the rats were returned to its original cage, afforded free access to food and water, and the survival was recorded every 12 h for 14 days. At the end of survival experiments, the rats were deeply anesthetized with pentobarbital sodium (100 mg/kg) and sacrificed. In separate experiments, rats were treated as described above, but a single dose of Ber (60 mg/kg) was used. Then, the cardiac function was assessed at 84 h after DOX administration with VisualSonics^R^ Vevo770™ High-Resolution In Vivo Imagine System ((VisualSonics Inc., Toronto, Ontario, Canada) as described previously [17]. After echocardiographic assessment, the rats were immediately anesthetized with pentobarbital sodium (50 mg/kg, intraperitoneally) and morphine hydrochloride (50 mg/kg, subcutaneously), the adequacy of the anesthesia was monitored by disappearance of the corneal reflex, loss of the pedal reflex and failure to respond to a skin incision. Then, the rats were sacrificed, the heart was rapidly excised, cardiac histological change, myocardial apoptosis and apoptosis-associated parameters were detected. Meanwhile, serum levels of LDH were measured by biochemistry autoanalyser.

**Figure 6:**
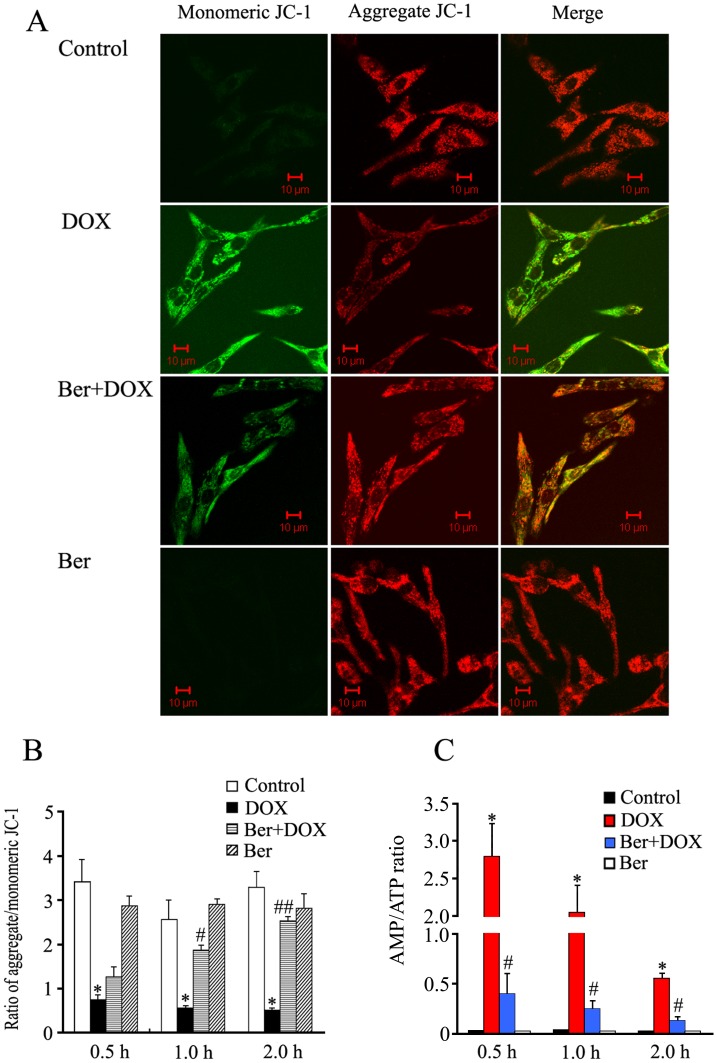
Effects of Ber (1.0 µM) on DOX (1.0 µM) - induced a decrease in mitochondrial membrane potential and a rise in AMP/ATP ratio in neonatal rat cardiomyocytes. (A) Confocal images of JC-1 fluorescence. Mitochondrial membrane potential of the cardiomyocytes was measured by JC-1, an indicator mitochondrial function, in cardiomyocytes treated with Ber (1.0 µM) or/and DOX (1.0 µM) for 1 h, red fluorescence represents the mitochondrial aggregate JC-1and green fluorescence indicates the monomeric JC-1. (B) Graph represents the ratio of aggregated and monomeric JC-1, indicating changes in mitochondrial membrane potential (n = 5). *P<0.001 compared with control group. ^#^ *P*<0.05, ^# #^ *P*<0.01 compared with DOX group. (C) Changes in AMP/ATP ratio in cardiomyocytes treated with Ber (1.0 µM) or/and DOX (1.0 µM) for 0.5 h, 1 h or 2 h (n = 3). *P<0.05 compared with control group. ^#^ *P*<0.05 compared with DOX group.

### Echocardiography Determination

Rats were anesthetized with 1.5% isoflurane (Rhodia UK LTD., Avonmouth, Bristol BS119YF, UK) and imaged in the supine position using a 17.5-MHz center frequency RMV 707 scanhead. Two-dimensional B-mode and M-mode imaging were acquired by a technician who was blinded to rat treatment, left ventricular (LV) end-diastolic and end-systolic diameters were measured on the M-mode parasternal short-axis tracing at papillary muscle level. Left ventricular end diastolic volume (LVEDV), stroke volume (SV) and LV ejection fraction (EF) were calculated with analysis software, and the data were averaged from at least three consecutive cardiac cycles.

### Histological Analysis

Cardiac tissues were fixed in 10% buffered formalin and embedded in paraffin. The paraffin sections were stained with hematoxylin and eosin for histological analysis under an optical microscopy.

**Figure 7:**
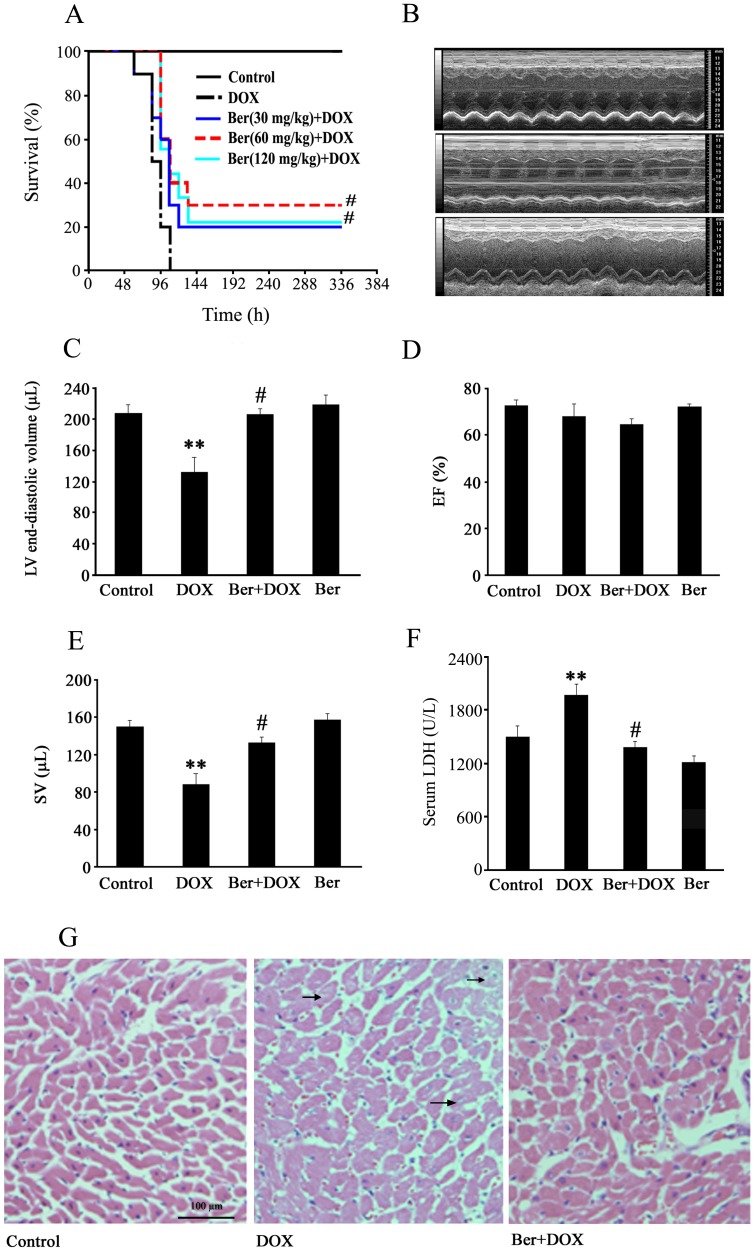
Effect of Ber on survival, cardiac function and myocardium injury in DOX-challenged rats. (A) Kaplan–Meier curves of survival in rats from control, 30 mg/kg Ber+20 mg/kg DOX, 60 mg/kg Ber+20 mg/kg DOX, 120 mg/kg Ber+20 mg/kg DOX and 20 mg/kg DOX groups, time is expressed in hours after the DOX injection (n = 9–10). (B) Representative M-mode echocardiographic images 84 h after DOX treatment in control (upper), 20 mg/kg DOX group (middle) and 60 mg/kg Ber+20 mg/kg DOX group (lower), and echocardiographic parameters (n = 6–8), including LVEDV (C), EF (D) and SV (E). (F) Changes in serum LDH activity 84 h after DOX treatment in control, 20 mg/kg DOX and 60 mg/kg Ber+20 mg/kg DOX and 60 mg/kg Ber groups (n = 7). (G) Representative photomicrographs of rat myocardium 84 h after DOX treatment in control, 20 mg/kg DOX and 60 mg/kg Ber+20 mg/kg DOX groups. The myocardium sections were stained with hematoxylin and eosin, and arrows indicate vacuolization. **P<0.01 compared with control group. ^#^ *P*<0.05 compared with DOX group.

### Apoptosis Assay

For apoptosis analysis, DNA fragmentation was determined by a Cell Death Detection ELISA kit (Roche Diagnostics Corporation, Indianapolis, IN, USA). Neonatal rat cardiomyocytes were cultured (2×10^4^ cells/well) for 2 days prior to the indicated treatments. At 24 h after DOX, Ber or/and vehicle treatment, the cytoplasmic mono- and oligonucleosomes (histone/DNA fragments) were extracted and assayed according to the manufacturer’s instructions. Absorbance was measured at 405 nm using a microplate reader. The specific enrichment of mono- and oligonucleosomes was expressed as enrichment factor and calculated using the following formula: enrichment factor = the ratio of absorbance of the sample to absorbance of negative control group.

**Figure 8:**
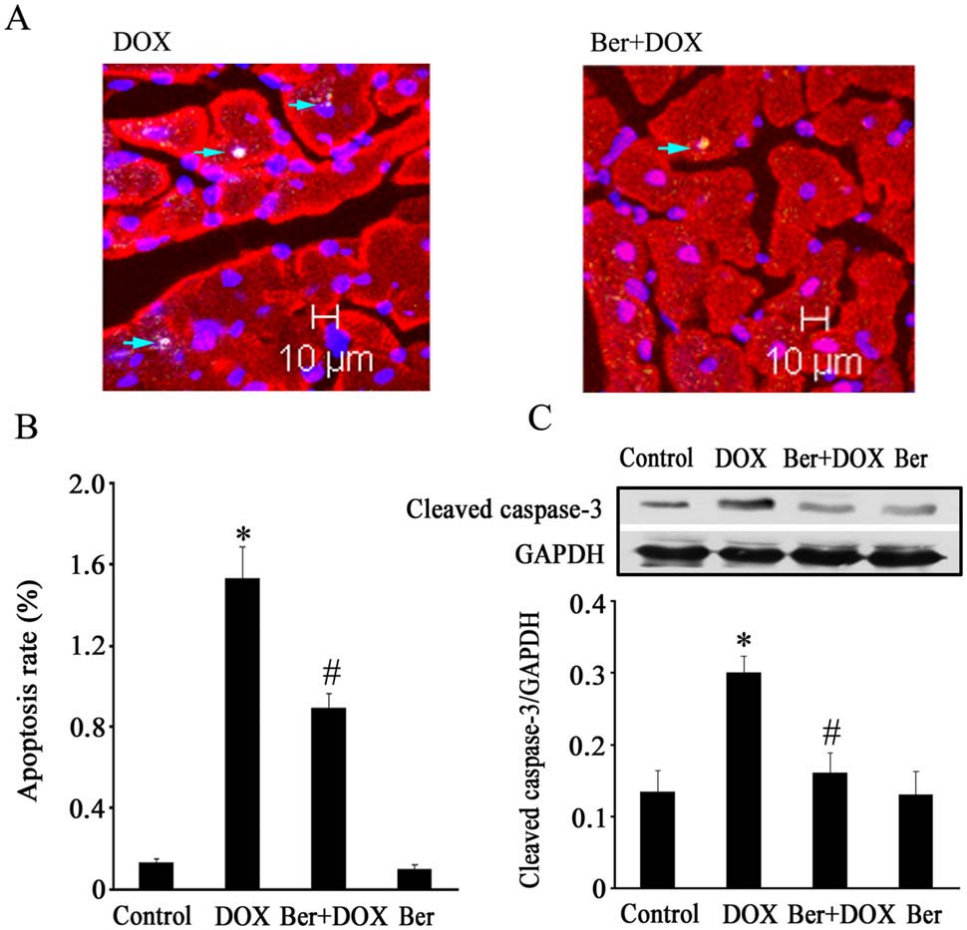
Effect of Ber on myocardial apoptosis in DOX-challenged rats. (A) Assessment of myocardial apoptosis *in vivo* by TUNEL staining 84 h after DOX (20 mg/kg) treatment in DOX and Ber (60 mg/kg)+DOX groups. Bars, 10 µm. Arrows indicate TUNEL-positive nuclei. (B) The myocardial apoptosis rate (n = 6). (C) Western blotting analysis of Cleaved caspase-3 in myocardium 84 h after DOX or vehicle treatment from control, 20 mg/kg DOX and 60 mg/kg Ber+20 mg/kg DOX and 60 mg/kg Ber groups (n = 6). *P<0.05 compared with control group. ^#^ *P*<0.05 compared with DOX group.

The terminal deoxynucleotidyl transferase-mediated dUTP nick end-labelling (TUNEL) assay was also carried out using an In-Situ Cell Death Detection Kit (Roche Diagnostics Corporation, Indianapolis, IN, USA). Neonatal rat cardiomyocytes were cultured on glass slides for 48 h. Cardiomyocytes were treated with compound C, DOX, Ber or/and vehicle for 24 h, then fixed in 95% ethanol at room temperature for 30 min, and permeabilized in 0.1% Triton-X 100. After blocking with normal goat serum for 30 min at room temperature, the cardiomyocytes were subjected to immunofluorescent staining with a mouse anti-cardiac troponin I (cTnI) antibody (1∶500 dilution, Abcam Inc., Cambridge, MA, USA), and DAPI (4,6-diamidi-no-2-phenylindole, Dojindo Molecular Technologies, Inc., Japan), followed by TUNEL reactions according to the manufacturer’s protocol. In *in vivo* experiments, the hearts were kept in cold normal saline to remove the blood, and fixed in ice-cold 4% paraformaldehyde dissolved in phosphate-buffered saline over night, then dehydrated and frozen in optimum cutting temperature compound at −80°C. The heart was sectioned at 10 µm, and the samples were then treated as described above. With confocal laser-scanning microscope (LSM510META, Zeiss, Germany), TUNEL-positive cardiomyocytes were counted in 5 randomly selected fields per section. The rate of apoptosis was expressed as the ratio of TUNEL-positive cardiomyocyte nuclei (green) to total number of cardiomyocyte nuclei (blue).

### Caspase- 3, 8 and 9 Activity Assessments

To measure caspase-3, 8 and 9 enzymatic activities, neonatal rat cardiomyocytes were cultured for 48 h followed by treatment with DOX (1 µM ) for 12 h in the presence or absence of Ber (1 µM). APO LOGIX Carboxyfluoroscein Caspase Detection Kits (Cell Technology Inc, Mountain View, CA, USA) were used to detect active caspase-3, 8 and 9 according to the manufacturer’s instructions.

**Figure 9:**
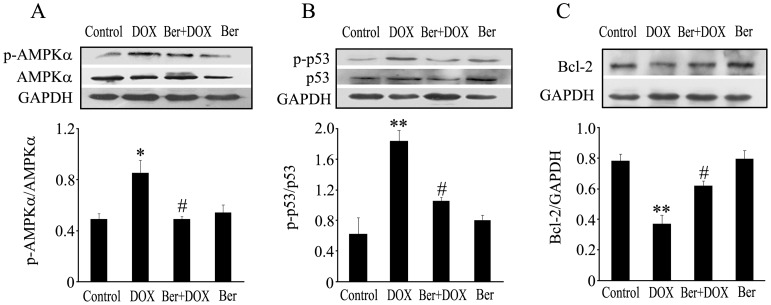
Changes of myocardial AMPKα phosphorylation, p53 phosphorylation and Bcl-2 protein level 84 h after DOX (20 mg/kg) or vehicle injection in rats from control, 20 mg/kg DOX and 60 mg/kg Ber+20 mg/kg DOX and 60 mg/kg Ber groups (n = 6–7). AMPKα phosphorylation (A), p53 phosphorylation (B) and Bcl-2 protein (C) were detected using Western blotting analysis. *P<0.05, **P<0.01 compared with control group. ^#^ *P*<0.05 compared with DOX group.

### Western Blotting

Neonatal rat cardiomyocyte or heart homogenates were harvested in RIPA buffer (PBS, 1% NP-40, 0.5% sodium deoxycholate, 0.1% SDS) containing 1 mM PMSF and incubated for 30 min on ice, then centrifuged at 4°C with 12000×*g* for 15 min. The supernatant lysates were diluted in 2× or 5×SDS sample buffer, boiled for 5 min. Unless otherwise mentioned, whole cell or tissue lysates were used for analysis. Mitochondrial proteins and cytosolic proteins were prepared using mitochondria isolation kit (Thermo scientific, Rockford IL, USA). Equal amounts of protein from each sample were resolved on SDS-polyacrylamide gel by electrophoresis, and transferred to immobilon polyvinylidene diflouride (PVDF, Millipore, Billerica, MA, USA) or NC(Nitrocellulose, Millipore, Billerica, MA, USA) membrane, blocked with 5% BSA in TBST (20 mM Tris–HCl, 137 mM NaCl, and 0.1% Tween 20, pH 7.5) at room temperature for 1 h. The membranes were incubated with primary anti-bodies over night at 4°C. Following incubation with HRP-conjugated secondary antibodies, then immunoblots were exposed on film with an enhanced chemiluminescence reagent (Thermo Scientific, Rockford IL, USA). The bands were quantified by optical density ratio using glyceraldehyde-3-phosphate dehydrogenase (GAPDH) or voltage-dependent anion channel (VDAC) as a control. The primary antibodies included Bcl-2, Cytochrome c, Bax, cleaved caspase-3, VDAC, GAPDH, p53, phospho-p53(Ser-15), AMPKα, phospho-AMPKα (Thr172), acetyl-CoA carboxylase (ACC) and phospho-ACC, all of which were purchased from Cell Signaling Technology, Inc. (Danvers, MA, USA).

### Measurement of Mitochondrial Membrane Potential

Neonatal rat cardiomyocytes were treated with Ber at concentration of 1.0 µM or vehicle for 20 min, and then exposed to 1.0 µM DOX for 30 min, 1 h or 2 h. Mitochondrial membrane potential was visualized in cardiomyocytes stained with 5, 5′, 6, 6′-tetrachloro-1, 1′, 3, 3′-tetraethylbenzimidazolcarbocyanine iodide (JC-1) by using a laser scanning confocal microscope. JC-1 is a dual-emission mitochondrial membrane potential sensing dye, which can be accumulated and aggregates in polarized (normal) mitochondria, and aggregate JC-1 shifts its emission to red. The mitochondrial depolarization (loss of mitochondrial membrane potential) prevent JC-1 entry into mitochondria, monomeric JC-1 remains in the cytosol and fluoresces green. Cardiomyocytes from different treatment groups were washed and incubated with JC-1 at 37°C for 15 min, then washed and mounted on the confocal microscopy for imaging. The aggregate JC-1 (red fluorescence) was detected at the emission wavelength of 590 nm, and the monomeric JC-1(green fluorescence) monitored at 529 nm. The ratio of aggregated and monomeric JC-1 was used to quantify changes in mitochondrial membrane potential, and decreased JC-1 ratio represented depolarization of the mitochondria indicating the decrease in mitochondrial membrane potential.

**Figure 10:**
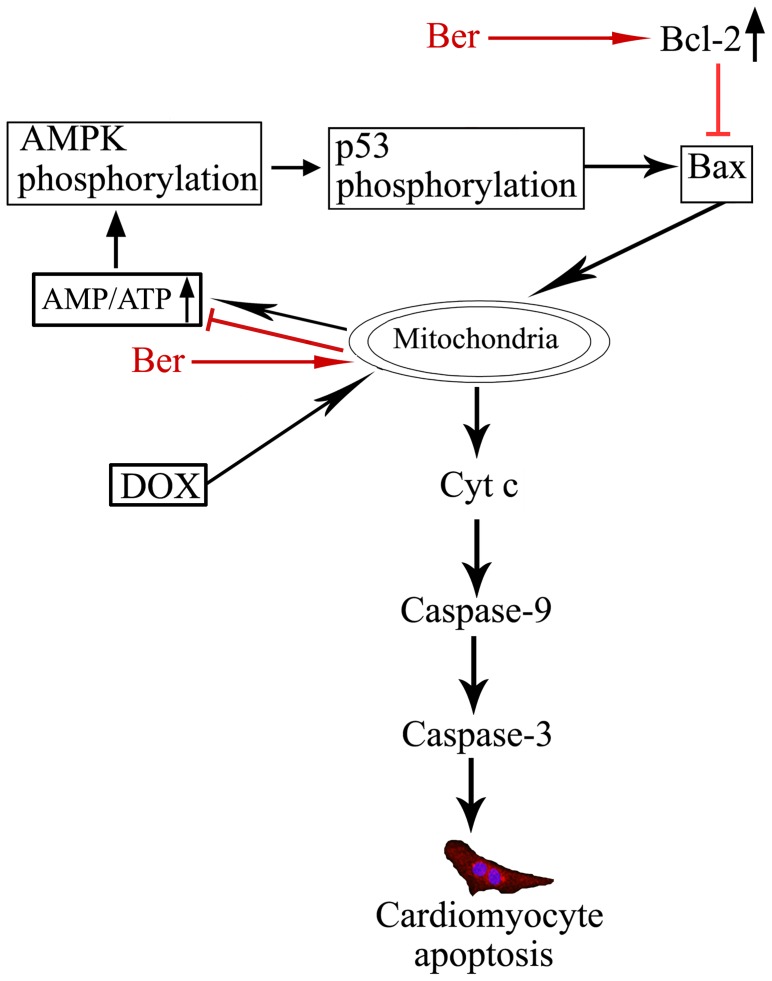
Summary of mechanisms responsible for inhibition of DOX-induced cardiomyocyte apoptosis by Ber. DOX directly causes mitochondrial injury, leads to a loss of mitochondrial membrane potential and a rise in AMP/ATP ratio, which induces AMPK and p53 phosphorylation, and then facilitates cardiomyocyte apoptosis. Ber inhibits DOX-induced apoptosis not only by upregulating Bcl-2 expression, but also by protecting mitochondria, reducing the AMP/ATP ratio and in turn suppressing AMPK phosphorylation.

### Measurement of Intracellular AMP and ATP Contents by High Performance Liquid Chromatography

Cardiomyocytes were extracted by adding 7% perchloric acid. The extracts were centrifuged at 12,000 *g* for 15 min at 4°C. Samples were neutralized with ice-cold 1 M K_2_HPO_4_ and analyzed by high performance liquid chromatography (HPLC)_._ HPLC was performed on a Boston 250×4.6 mm C_18_ column. The flow rate was 1 ml/min at 25°C with a starting buffer of 50 mM potassium phosphate buffer (pH 6.5). The injection volume was 40 µL. Peaks were identified by comparison with retention times of standards and analysis of peak spectra from a recorded 3d-field with Chromeleon™ software. Adenosine triphosphate (ATP) and adenosine monophosphate (AMP) were determined by measuring peak area at 256 nm. The contents of AMP and ATP in samples were calculated according to standards of known concentration and expressed as nM/mg protein.

### Statistical Analyses

Quantitative data are expressed as mean±standard error of the mean (SEM). Statistical differences among groups were evaluated with one-way analysis of variance (ANOVA) followed by Bonferroni post hoc analysis. Mortality was analyzed using Kaplan-Meier survival analysis and compared by log-rank test. Differences were considered statistically significant at *P*<0.05.

## Results

### Ber Protects Cardiomyocytes against Injury Induced by DOX *in vitro*

To determine whether Ber protects cardiomyocytes against DOX-induced injury, the damage of neonatal rat cardiomyocytes was assessed by measuring LDH activity in the culture medium of cardiomyocytes. In the presence of 1.0 µM DOX for 24 h, the LDH activity in culture medium for the DOX-treated cardiomyocytes was significantly higher than that for control cells (*P*<0.01). However, treatment with Ber at doses of 0.25, 1.0 and 4.0 µM attenuated DOX-induced increase in LDH activity (*P*<0.01, respectively). Ber alone has no obvious effect on LDH activity in the culture medium of cardiomyocytes (Figure 1A). In addition, various concentrations of Ber have no significant effect on the antitumor activity of DOX in MCF-7 cells (Figure 1B).

### Ber Inhibits DOX-induced Neonatal Rat Cardiomyocyte Apoptosis

Apoptosis was determined by an ELISA assay to quantify histone-associated DNA fragmentation generated in apoptotic cardiomyocyte. As depicted in Figure 2A, 1 µM DOX treatment for 24 h resulted in a noticeable increase in DNA fragmentation. In contrast, Ber (0.25–4.0 µM) reduced DNA fragmentation in DOX-stimulated cardiomyocytes in a dose-dependent manner.

Apoptotic cells were also detected by TUNEL assay (Figure 2B). As shown in Figure 2C, exposure to 1.0 µM DOX for 24 h caused a significant increase in cardiomyocyte apoptosis rate compared with control. After treatment with 0.25, 1.0 and 4.0 µM Ber, apoptosis rate was decreased in cardiomyocytes stimulated with DOX (*P*<0.05, 0.01).

### Mitochondrial Pathway is Involved in Inhibitory Effect of Ber on Neonatal Rat Cardiomyocyte Apoptosis Induced by DOX

It has been reported that DOX-induced apoptosis of cardiomyocytes is a multifactorial process [7]. We examined the activities of caspases-3, 8 and 9 as well as cytosolic cytochrome c, Bcl-2 and mitochondrial Bax levels in neonatal rat cardiomyocytes. As shown in Figure 3, caspase-3 activity was dramatically higher 12 h after DOX treatment in the DOX group than that in control. After the addition of 1.0 µM Ber, caspases-3 activity depressed markedly in DOX+Ber group compared with DOX group (Figure 3A). Caspases-9 activity showed a significant increase 12 h after 1.0 µM DOX exposure, which was almost completely attenuated in the presence of 1.0 µM Ber (Figure 3C). However, the activity of caspase-8 had no evident change 12 h after DOX or/and Ber stimulation (Figure 3B). Moreover, exposed to 1.0 µM DOX for 6 h, cytoplasmic cytochrome c and mitochondrial Bax levels significantly increased compared with control. In contrast, cytoplasmic cytochrome c and mitochondrial Bax levels were attenuated by Ber treatment in DOX-treated cardiomyocytes (Figure 3D and 3E). Meanwhile, DOX (1.0 µM) reduced Bcl-2 expression in cardiomyocytes, which was significantly elevated by Ber treatment (Figure 3F).

### AMPK and p53 Signal Pathways Mediate Inhibitory Effect of Ber on DOX-induced Apoptosis in Neonatal Rat Cardiomyocytes

Previous studies have demonstrated that DOX induces AMPK and p53 activation leading to cardiomyocyte apoptosis [10]–[12]. Thus, we examined phosphorylated p53 (p-p53), p53, AMPKα and phosphorylated AMPKα (p-AMPKα) protein levels in neonatal rat cardiomyocytes. The p-p53 level was markedly increased in DOX group compared with control, but decreased in Ber+DOX group compared with DOX group 2 h after DOX treatment (Figure 4A). The same tendency was observed in p-AMPKα (Figure 4B). Furthermore, compound C largely inhibited AMPKα phosphorylation and in turn p53 phosphorylation, but did not affect the inhibitory effect of Ber on AMPKα and p53 phosphorylation in DOX-challenged cardiomyocytes (Figure 4C and 4D), and compound C also inhibited DOX-induced cardiomyocyte apoptosis (Figure 4E).

In order to further investigate the effect of DOX and Ber on AMPKα phosphorylation in neonatal cardiomyocytes, we determined the phosphorylation level of ACC, a downstream target of AMPK, as well as the effects of DOX and Ber at varying doses or different exposure time on AMPKα phosphorylation. Cardiomyocytes were pretreated with AMPK activator, AICAR (0.5 mM), or vehicle for 90 min, and then incubated with DOX (1.0 µM) or/and Ber (1.0 µM) for 2 h, the ACC phosphorylation was measured by Western blotting. As shown in Figure 5A, DOX and AICAR significantly increased ACC phosphorylation compared with control (*P*<0.01); Ber markedly suppressed DOX-stimulated ACC phosphorylation (*P*<0.05), but not AICAR-induced ACC phosphorylation; DOX did not affect AICAR-induced ACC phosphorylation. In addition, cardiomyocytes were exposed to 1.0 µM DOX for 12 h (Figure 5B) or 24 h (Figure 5C), AMPKα phosphorylation still increased compared with control, which was also inhibited by 1.0 µM Ber treatment. Moreover, DOX (0.5–2.0 µM) or Ber (16.0 µM) exposure for 2 h markedly caused AMPKα phosphorylation (Figure 5D and 5E), but Ber at a concentration of 0.25, 1.0 or 4.0 µM did not significantly stimulate AMPKα phosphorylation (Figure 5E).

### Ber Reduces DOX-induced Mitochondrial Membrane Potential Loss and a Rise in the AMP/ATP Ratio in Neonatal Rat Cardiomyocytes

As shown in Figure 6A and 6B, exposure of neonatal rat cardiomyocytes to DOX (1.0 µM) for 30 min, 1 h or 2 h all induced a marked decrease of mitochondria membrane potential, and Ber reduced DOX-induced mitochondria membrane potential loss in a time-dependent manner. DOX exposure for 30 min, 1 h or 2 h also increased the AMP/ATP ratio in neonatal rat cardiomyocytes, all of which were inhibited by Ber treatment (Figure 6C).

### Ber Improves Survival, Increases Cardiac Stroke Volume and Attenuates Myocardial Injury in DOX-challenged Rats

Figure 7A showed that survival rate was significantly lower in DOX group than that in control group. In contrast, the survival rate was significantly higher in Ber (60 mg/kg)+DOX group and Ber (120 mg/kg)+DOX group than that in DOX group (*P*<0.01, n = 9–10*).* All rats survived at 72 h after DOX challenge in Ber (60 mg/kg)+DOX group.

Echocardiographic examination demonstrated that DOX treatment significantly reduced LVEDV and SV at 84 h in rats. In contrast, LVEDV and SV were higher in Ber (60 mg/kg)+DOX group than those in DOX group (Figure 7C and 7E). However, treatment with DOX or/and Ber had no obvious effect on left ventricular EF at 84 after DOX challenge (Figure 7D).

Histological examination of cardiac sections stained with hematoxylin-eosin showed that DOX induced cardiomyocyte cytoplasmic vacuolization, an essential feature of DOX cardiotoxicity, which was attenuated by Ber treatment (Figure 7G). As shown in Figure 7F, the serum LDH activity 84 h after DOX treatment in DOX group was significantly higher than that in control (*P*<0.01) and Ber+DOX groups (*P*<0.05).

### Ber Inhibits Myocardial Apoptosis in DOX-challenged Rats

As shown in Figure 8, the number of TUNEL-positive cardiomyocytes increased 84 h after DOX injection in DOX group, but decreased in Ber (60 mg/kg)+DOX group (Figure 8A and 8B) compared with DOX group (*P*<0.05). Moreover, the level of cleaved caspase-3 protein in myocardium was higher at 84 h after DOX challenge in DOX group than that in control. This alteration of cleaved caspase-3 protein expression induced by DOX was reversed by Ber (60 mg/kg) treatment (Figure 8C).

### Ber Suppresses Myocardial AMPKα and p53 Phosphorylation and Increases Bcl-2 Protein Expression in DOX- challenged Rats

To confirm our findings that inhibition of AMPKα and p53 phosphorylation contributes to the cardioprotection of Ber *in vitro*, we further detected myocardial AMPKα, p-AMPKα, p53, p-p53 and Bcl-2 protein levels by western blot assay in rats. The ratio of p-AMPKα to AMPKα and the ratio of p-p53 to p53 increased 84 h after DOX injection in DOX-challenged rats, but AMPKα and p53 phosphorylation degree were decreased in Ber (60 mg/kg)+DOX group compared with DOX group (Figure 9A and 9B). On the contrary, the expression of myocardial Bcl-2 protein was reduced 84 h after DOX injection in DOX group compared with control, but was elevated in Ber (60 mg/kg)+DOX group compared with DOX group(Figure 9C).

## Discussion

DOX-induced cardiomyopathy remains a lethal disease. Unfortunately, the clinically effective therapy and preventive treatment are yet to be discovered [2]. The present study is the first to show that Ber inhibits DOX-triggered cardiomyocyte apoptosis *in vitro* and *in vivo*.

Numerous researches have demonstrated that Ber possesses antitumor activity and induces apoptosis in many cancer cell lines in a concentration-dependent manner [18], [19]. Conversely, Zhou et al. recently found that 1 to 5 µM Ber markedly inhibited apoptosis of PC12 cells induced by oxygen-glucose deprivation [20]. Wang et al. also observed that Ber (25 µM) significantly prevented hyperglycemia-induced endothelial apoptosis [21]. These investigations indicate that the effect of Ber on apoptosis is concentration- and cell type - dependent. In clinical practice, the dose of Ber given to patient was often controlled within 0.3–0.6 g every time and its peak concentration in the plasma could reach 1–2 µM [22]. Zeng et al. found that Ber at plasma concentrations more than 0.27 µM significantly improved the symptoms of patients with congestive heart failure [23]. Accordingly, we examined the effect of Ber at concentrations of 0.06, 0.25, 1.0 or 4.0 µM on cultured neonatal rat cardiomyocyte injury in the present study, and found Ber concentration-dependently reduced cell damage in cardiomyocytes exposed to 1.0 µM DOX, evidenced by decreased LDH release. Interestingly, Ber at dose of 0.25, 1.0 and 4.0 µM did not significantly affect the viability of breast cancer MCF-7 cells treated with 1.0 µM DOX. These findings suggest that Ber could protect against cardiotoxicity without impairing the anti-tumor efficiency produced by DOX. Furthermore, Ber markedly alleviated cardiomyocyte apoptosis induced by DOX in a dose-dependent manner as evidenced by TUNEL and DNA fragmentation assay. It has demonstrated that DOX-induced cardiomyocyte apoptosis involves the extrinsic and intrinsic pathways [7]. Thus, we examined the effects of Ber on the activities of caspase-8, caspase-9 and caspase-3 as well as cytosolic cytochrome c level in neonatal rat cardiomyocytes treated with DOX. The results showed that Ber at a dose of 1.0 µM significantly decreased caspase-9 and capase-3 activities, but not caspase-8 activity in cardiomyocytes stimulated with DOX. DOX stimulation induced an increase in cytosolic cytochrome c level, which was attenuated by Ber treatment. These results suggest that Ber inhibited cardiomyocytes apoptosis induced by DOX might be associated with mitochondria signaling pathway. However, Ber almost completely suppressed DOX-induced caspase-9 and capase-3 activation. As shown in Figure 2, Ber partly decreased, but not completely blocked DOX-induced cardiomyocyte apoptosis. One possibility for this phenomenon is that caspase-3-independent mechanisms are involved in DOX-induced cardiomyocyte apoptosis, as some studies have demonstrated that caspase-12 activation mediates DOX-provoked cardiomyocyte apoptosis [24], [25].

It has been proved that Bax translocation from the cytosol to mitochondria results in cytochrome c release from the mitochondria, anti-apoptotic Bcl-2 can interfere with mitochondrial cytochrome c release and suppress apoptosis progression [26], [27]. We further observed the effect of Ber on Bax translocation and Bcl-2 expression in DOX-stimulated cardiomyocytes. Western blot assay showed that DOX markedly increased mitochondrial Bax level and decreased Bcl-2 expression, and these effects of DOX were inhibited by Ber treatment, suggesting that Ber attenuates DOX-induced neonatal cardiomyocyte apoptosis via inhibiting Bax translocation to mitochondria and upregulating Bcl-2 expression.

Numerous studies have demonstrated that DOX-induced cardiomyocyte apoptosis is associated with activation of p53 tumor suppressor protein [10], [13], [28], [29]. DOX can accumulate in the cardiomyocyte nucleus and interfere with DNA, resulting in p53 overexpression and phosphorylation on Ser-15 (activated). This activation of p53 is linked to the down-regulation of Bcl-2 and up-regulation of Bax following with activating the intrinsic apoptosis pathway [30], [31]. Consistent with these studies, we also found that DOX stimulated p53 phosphorylation on Ser-15 and increased p53 expression in neonatal rat cardiomyocytes, Ber abrogated the expression and phosphorylation of p53 induced by DOX. Therefore, our observations indicate that mechanisms underlying inhibition of DOX-induced cardiomyocyte apoptosis by Ber are associated with suppressing p53 overexpression and phosphorylation.

More recently, it was reported that AMPK activation induced p53-dependent apoptosis [32]. DOX treatment for 2 h induced AMPKα (Thr172) phosphorylation in cardiomyocytes. Both AMPK inhibitor, compound c, and AMPKα siRNA largely inhibited DOX-induced p53 activation (Ser 15 phosphorylation and expression), Bax up-regulation, Caspase 3 cleavage and cardiomyocyte apoptosis. These results indicate that AMPKα activation contributes to DOX-induced cardiomyocyte apoptosis through inducing phosphorylation of p53 on Ser-15 [12]. In the present study, we also observed that DOX remarkably elevated phosphorylation of AMPKα and p53 at 2 h after DOX stimulation in neonatal cardiomyocytes, and compound C, an inhibitor of AMPK phosphorylation, suppressed DOX-provoked AMPKα phosphorylation and in turn p53 phosphorylation and cardiomyocyte apoptosis. However, compound C did not completely reversed DOX-provoked cardiomyocyte apoptosis. DOX has been reported to induce cardiomyocyte apoptosis through its activation of a variety of signaling pathways, including oxidative stress, calcium, transcription factors and mitogen-activated protein kinases [4], [7]. Thus, the current results confirm that AMPKα activation facilitates, at least in part, DOX-induced cardiomyocyte apoptosis. Particularly, we further found that Ber diminished the phosphorylation of AMPKα and p53 stimulated by DOX. In order to confirm the effect of Ber on DOX-induced AMPKα phosphorylation in cardiomyocytes, we examined the phosphorylation of ACC, a downstream target of AMPK, using an AMPK activator, AICAR, as a positive control. The results demonstrated that DOX and AICAR significantly increased ACC phosphorylation and Ber markedly suppressed DOX-stimulated ACC phosphorylation in cardiomyocytes. Based on the above data, we conclude that Ber protects against DOX-induced cardiomyocyte apoptosis by inhibiting AMPKα and p53 phosphorylation.

However, some researchers reported that DOX inhibited AMPKα phosphorylation in cardiomyocytes [33], [34], In these studies, AMPK phosphorylation was detected at 24 h and 16 h after DOX stimulation, whereas AMPK and p53 phosphorylation were examined at 2 h after DOX exposure in our study. In fact, they also observed that DOX activated AMPK at the initial stage (<4 h) [34]. We further investigated the effects of DOX at varying doses or different exposure time on AMPKα phosphorylation in neonatal rat cardiomyocytes. The results showed that DOX exposure for 2 h caused AMPKα phosphorylation in a dose-dependent manner. Moreover, exposure of cardiomyocytes to 1.0 µM DOX for 24 h still increased AMPKα phosphorylation, which is inconsistent with the previous studies [33], [34]. These contradictory findings might be due to different treatment time and cell types as H9C2 cardiomyocytes, neonatal mouse cardiomyocytes cultured for 24 h or neonatal rat cardiomyocytes cultured for 48 h were used, respectively, in these investigations. On the other hand, Ber has also been demonstrated to induce AMPK phosphorylation in skeletal muscles and endothelial cells, and activation of AMPK by Ber prevented hyperglycemia-induced endothelial apoptosis [21], [35]. It is important to note that concentrations of Ber used in these studies were more than 5.0 µM, while 0.25–4.0 µM Ber were used in the present study. Furthermore, we observed that treatment with Ber for 2 h induced AMPKα phosphorylation in a dose-dependent manner, but Ber at a dose of 0.25, 1.0 or 4.0 µM did not significantly increase AMPKα phosphorylation. Therefore, different concentrations of Ber play distinct roles in various cell lines.

More importantly, both DOX and AMPK activator, AICAR, significantly increased phosphorylation of AMPK downstream target ACC; Ber markedly suppressed DOX-stimulated ACC phosphorylation, but not AICAR-induced ACC phosphorylation; Additionally, DOX did not affect AICAR-induced ACC phosphorylation. Thus, DOX or Ber itself may not directly affect AMPK phosphorylation in the present study and inhibition of DOX-induced AMPK phosphorylation by Ber in cardiomyocytes at 2 h after DOX exposure may involve other initial events. It is well known that the increase in the ratio of intracellular AMP to ATP is a major regulator of AMPK activity [36]. On the other hand, it has been demonstrated that cardiolipin in the inner mitochondrial membrane can concentrate DOX [37], Pointon et al. found that DOX caused myocardial mitochondria dysfunction and a rapid fall in cardiomyocyte adenosine triphosphate (ATP) content, increased the ratio of AMP to ATP, activated AMPK and elevated caspase-3 activity over the time course from 30 to 120 min following acute DOX exposure in mice [38]. It is for this reason that we further investigated the effect of Ber on mitochondrial membrane potential and the ratio of AMP to ATP in DOX-treated cardiomyocytes at 30 min, 1 h and 2 h after DOX exposure, respectively. The results showed that DOX induced a rapid decrease in mitochondrial membrane potential and a rise in the ratio of AMP to ATP in cardiomyocytes at 30 min, 1 h and 2 h after DOX exposure. Ber inhibited DOX-induced mitochondrial membrane potential loss and the increase in the ratio of AMP to ATP. The previous study demonstrated that Ber was found to be concentrated in mitochondria at low doses [39]. Accordingly, these data suggest that Ber reduces DOX-stimulated AMPKα phosphorylation in cardiomyocytes at early stage after DOX exposure via protecting mitochondria and reducing the increased ratio of intracellular AMP to ATP. Taken in conjunction, the current study indicates that Ber inhibits DOX-induced neonatal rat cardiomyocyte apoptosis via protecting mitochondria, reducing the increased ratio of AMP to ATP and in turn suppressing AMPKα phosphorylation. However, it is worth to note that adiponectin is found to upregulate AMPK expression, induce its phosphorylation, and partially suppress cardiomyocyte apoptosis caused by DOX in a recent study [33]. In their study, cardiomyocytes were pretreated with exogenous adiponectin. It is likely that adiponectin-induced AMPK activation might increase the ATP level in cardiomyocytes. In this condition, DOX challenge might be unable to rapidly decrease ATP content, increase the ratio of AMP to ATP, activate AMPK and facilitate cardiomyocyte apoptosis. In support of this notion, our study provided indirect evidence that pretreatment of cardiomyocytes with AMPK activator, AICAR, for 90 min increased ACC phosphorylation and DOX challenge after AICAR treatment did not affect AMPK downstream target ACC phosphorylation. Further studies are necessary to resolve these issue by detecting AMP and ATP levels in cardiomyocytes pretreated with AMPK activator and then with DOX.

Furthermore, we examined the effect of Ber on DOX-stimulated cardiomyocyte apoptosis in a rat model of acute DOX-induced cardiotoxicity. Our present results demonstrated that DOX administered at a single dose of 20 mg/kg increased mortality and serum LDH activity, induced myocardial injury and reduced LVEDV and SV at 84 h after DOX treatment. TUNEL and Western blotting analysis revealed that DOX significantly increased the number of TUNEL-positive cardiomyocytes and cleaved caspase-3 protein level in myocardium 84 h after DOX challenge, these effects of DOX were markedly reduced by Ber. These findings indicate that Ber improves survival and inhibits myocardial apoptosis and injury in an acute DOX-induced cardiotoxicity model, but the details of the mechanism by which DOX decreases LVEDV is unknown. However, DOX challenge did not reduce left ventricular EF 84 h after DOX challenge in the present study. It has demonstrated that elevated catecholamine can compensate for cytotoxic damage and preserve normal cardiac contraction in the early stages of DOX-induced cardiac dysfunction [40]. Thus, the reason for normal left ventricular EF at 84 h after DOX challenge might be related to the compensatory enhanced myocardial contractility resulted from sympathetic nervous activation. In order to confirm the *in vitro* observations, we also investigated cardiac AMPKα and p53 phosphorylation as well as Bcl-2 protein expression in DOX- challenged rats. The results showed that DOX- stimulated cardiac AMPKα and p53 phosphorylation were markedly attenuated by Ber. In addition, Ber enhanced myocardial Bcl-2 protein level in DOX-challenged rats. Therefore, the results from the *in vivo* experiments also indicate that Ber protects against DOX-induced cardiomyocyte apoptosis, at least in part, through inhibiting myocardial AMPKα and p53 phosphorylation and elevating Bcl-2 expression in the early stage of DOX treatment.

In summary, the present study provides evidence to show that DOX rapidly induces mitochondrial injury, increases the ratio of intracellular AMP to ATP, in turn activates AMPK phosphorylation and facilitates cardiomyocyte apoptosis. Ber suppresses DOX-induced cardiomyocyte apoptosis via protecting mitochondria, reducing the increased ratio of AMP to ATP and inhibiting AMPK phosphorylation as well as elevating Bcl-2 expression in the early stage of DOX treatment (Figure 10). Moreover, Ber also improves survival in an acute DOX-treated rat model. Therefore, we suggest that Ber is a promising, adjunctive therapeutic agent that prevents DOX-triggered cardiomyopathy without compromising its antitumor benefits. However, further studies must be carried out in a chronic DOX-induced cardiotoxicity model and in clinical trials to confirm how to use Ber in the treatment of DOX-triggered heart failure.
